# The Hox-TALE has been wagging for a long time

**DOI:** 10.7554/eLife.02515

**Published:** 2014-03-18

**Authors:** David EK Ferrier

**Affiliations:** 1 **David EK Ferrier** is at the Scottish Oceans Institute, Gatty Marine Laboratory, University of St Andrews, St Andrews, United Kingdomdekf@st-andrews.ac.uk

**Keywords:** Hox, TALE, evolutionary developmental biology, network, transcription factors, *Nematostella vectensis*, *D. melanogaster*

## Abstract

Hox and TALE proteins interact in a sea anemone, just as they do in flies and mice, indicating that the Hox-TALE system originated very early in animal evolution.

**Related research article** Hudry B, Thomas-Chollier M, Volovik Y, Duffraisse M, Dard A, Frank D, Technau U, Merabet S. 2014. Molecular insights into the origin of the Hox-TALE patterning system. *eLife* **3**:e01939. doi: 10.7554/eLife.01939

**Image** The starlet sea anemone occupies one of the early branches of the animal family tree

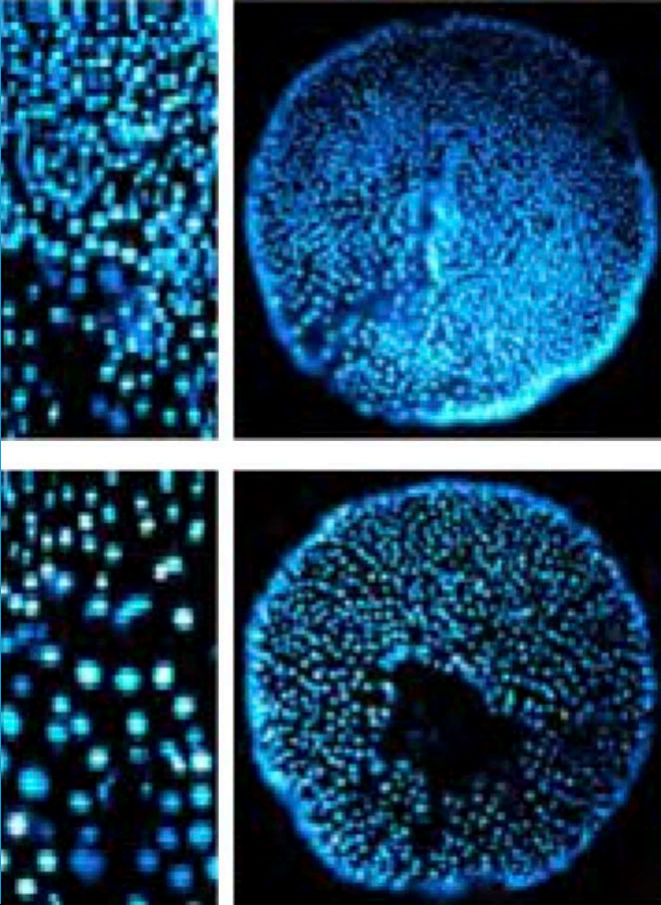

Animals come in many different shapes and sizes. Most of them—from worms and insects to fish and humans—are roughly symmetrical along a line that runs from the ‘head’ to the ‘tail’, and have a left side that mirrors the right side. However, there are notable examples of animals that do not show this bilateral symmetry, such as sponges and jellyfish. The origin of all of these animal forms, and ultimately ourselves, is entwined with the evolution of the developmental mechanisms that build animals. Moreover, many of the genes responsible for building humans are found in other animals, and they often do similar jobs in these different species. A good example is a subset of homeobox genes known as the ‘Hox genes’.

Hox genes are famous for often being found in clusters, with the order of the genes within the cluster matching the order in which these genes are first activated along the head-to-tail axis of the embryo. These genes code for Hox proteins that can interact with DNA to switch other genes ‘on’ or ‘off’. The number of different Hox proteins is relatively small, but they are able to target a wide spectrum of other genes, with their ability to bind to different target genes being modulated via interactions with other proteins known as co-factors. The ability of a relatively small number of Hox genes to specify the huge diversity of animal body forms observed in nature is a major puzzle in developmental biology.

Work on flies and mice has revealed that the major Hox co-factors belong to the so-called TALE class of homeobox genes (Holland et al., 2007). Now, in *eLife*, Bruno Hudry of Imperial College London, Samir Merabet of the Institut de Génomique Fonctionnelle de Lyon and co-workers have uncovered the origin of this co–factor interaction by focussing on an early branch of the animal family tree, the cnidarians, which includes jellyfish, corals and anemones (Hudry et al., 2014).

We know that TALE genes, specifically those belonging to the PBC/Pbx and Meis families of genes, evolved before the origin of animals because copies of TALE genes are clearly present in some of the single-cell relatives of the multicellular animals. However, it has been difficult to determine when Hox genes evolved relative to the origin of animals. Some researchers have proposed that Hox genes evolved coincidently with the origin of animals, and were then lost in some early animal lineages (Mendivil Ramos et al., 2012). Others have suggested that they originated somewhere within the animal kingdom, some time after the divergence of the very earliest branches, such as the sponges (Ryan et al., 2010). Nevertheless, Hox genes had evolved by the time the cnidarian and bilaterian lineages split from each other. A second major point of debate is whether the Hox genes of cnidarians function in the same fashion as those in bilaterians.

Now Hudry et al.—who are based in the UK, France, Israel and Austria—establish that Hox-TALE protein–protein interactions occur in the cnidarians. This reveals that the Hox genes of bilaterians and those of the early animal lineages, like the cnidarians, are more similar than previously recognised.

Hudry et al. show that although there are TALE genes in the single-celled relatives of the animals, they lack some of the sequence motifs that are needed to interact with Hox proteins, and cannot form protein–protein complexes with each other. Only in animal lineages that contain unambiguous Hox genes—the cnidarians and the bilaterians—do these proteins have all of the necessary motifs to form these complexes. Intriguingly, in the starlet sea anemone, *Nematostella vectensis*, Hox-TALE complexes containing different Hox proteins bind to distinct DNA sequences. This is comparable to the different target sequences that are bound by distinct bilaterian Hox-TALE complexes, which in turn correspond to distinct functions along the head-tail axis of bilaterian embryos. Furthermore, this ‘axial’ difference in the activities of Hox proteins from the starlet sea anemone is also evident in the degree to which they rescue a nervous system mutation in the bilaterian fly, *Drosophila melanogaster*. This is despite the contentious issue, discussed by Hurdy et al., as to whether the ‘mouth’ end of the cnidarian body plan corresponds to the head or tail end of a bilaterian.

Assembly of the Hox-TALE complex typically involves one TALE protein—a PBC/Pbx protein—binding to a hexapeptide motif (HX) in the Hox protein. Some other homeobox genes also encode proteins with HX motifs, including the Msx gene of *N. vectensis*. Hudry et al. show that the Msx protein also forms a complex with the TALE proteins, and that this requires the HX motif. However, the formation of these Hox-TALE and Msx-TALE complexes does not occur in the exact same way in *N. vectensis*, with the latter requiring another TALE protein, called Meis, to be present. Furthermore, although mutating the HX motif can block the Hox-TALE complex, the presence of Meis can restore the complex, which demonstrates that further interaction motifs, besides the HX, are used by Hox proteins. Also, HX motifs are found in several non-Hox proteins across the animal kingdom. As such, understanding the different ways that these protein complexes can form—which probably reflects the diversity of functions that they perform—is likely to be of widespread importance.

The Hox/Pbx/Meis complex, which is essential for directing various aspects of axial development in the vast majority of animals, appears to have evolved in a somewhat piece-meal fashion. It was established by the time of the cnidarian-bilaterian ancestor and constitutes a key system around which so much of the diversity in animal body forms subsequently evolved. Evolutionary diversity clearly abounds within and between these early branches of the animals, however, with different patterns of gene loss (Peterson and Sperling, 2007; Ryan et al., 2013; Riesgo et al., 2014) and HX motifs being absent from some cnidarian Hox proteins. Consequently, wider sampling is still needed to help establish whether the Hox-TALE interactions characterised by Hudry et al. really did originate with the cnidarian-bilaterian ancestor or if, in fact, they were established even earlier.

**Figure 1.**
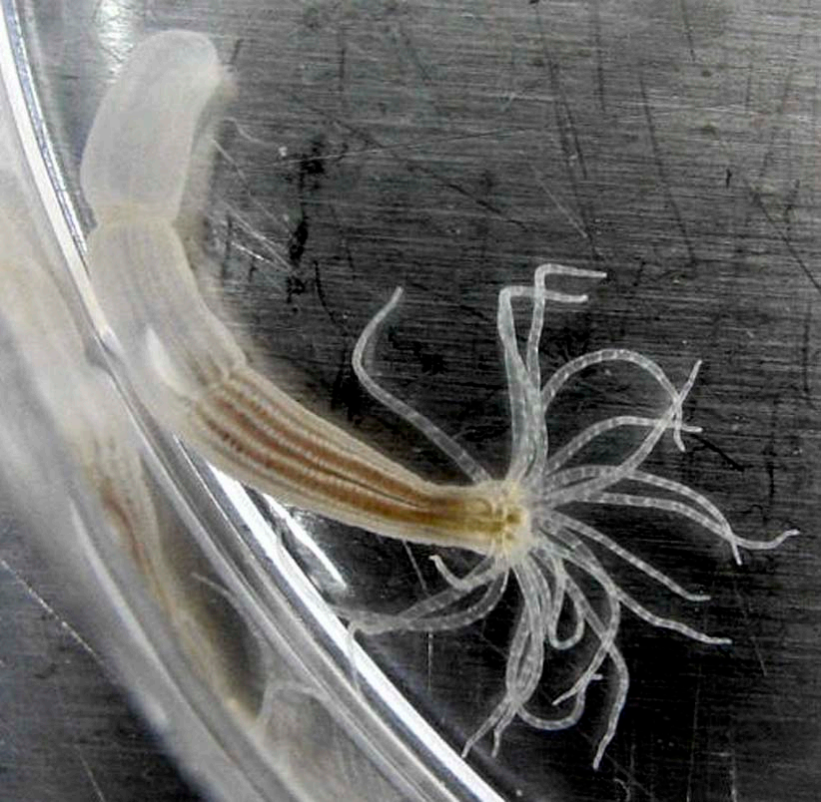
The starlet sea anemone. The cnidarians, such as the starlet sea anemone (*N. vectensis*) shown here, have a body form that is very different to the bilaterally symmetrical form found in most other animals. Anemones have a mouth surrounded by tentacles at one end and a foot that attaches to the substrate at the other. Hudry et al. have shown that, despite such a difference in general body form, the Hox-TALE system that operates in the development of cnidarians functions in a similar fashion to the Hox-TALE system of flies and mice.

Image: Cymothoa exigua.
